# Developmental changes of GABA immunoreactivity in cortico-thalamic networks of an absence seizure model

**DOI:** 10.1016/j.neuropharm.2018.01.047

**Published:** 2018-07-01

**Authors:** Cristiano Bombardi, Marcello Venzi, Vincenzo Crunelli, Giuseppe Di Giovanni

**Affiliations:** a University of Bologna, Department of Veterinary Medical Science, Bologna, Italy; b Neuroscience Division, School of Bioscience, Cardiff University, Museum Avenue, Cardiff CF10 3AX, UK; c Department of Physiology and Biochemistry, University of Malta, Malta

**Keywords:** Immunohistochemistry, Thalamus, Developmental GABAergic quantification, Epilepsy, Interneurons

## Abstract

Absence seizures (ASs) are associated with abnormalities in gamma-aminobutyric acid (GABA) neurotransmission in the thalamus and the cortex. In the present study, we used light microscopy GABA immunocytochemistry to quantify the GABA-immunoreactive (GABA-IR) neurons and neuropil in the thalamic ventral basal (VB) nucleus, the nucleus reticularis thalami (NRT), the dorsal lateral geniculate (dLGN), the primary motor cortex (M1) and perioral region of the somatosensory cortex (S1po) of genetic absence epilepsy rats from Strasbourg (GAERS). We used both the relative non-epileptic control (NEC) and normal Wistar rats as control strains, and investigated GABA immunostaining at postnatal day 15 (P15), P25, and P90. The main findings were i) an increase in GABA-IR neuropil in the VB at P25 and P90 in GAERS but not in NEC and Wistar rats; ii) an increase in NRT GABA-IR neurons in GAERS and NEC, but not Wistar, rats at both P25 and P90; and iii) an increase in GABA-IR neuron density in S1po of GAERS at P25 and P90 and in Wistar at P90. These results indicate that the increased GABAergic innervation in the VB at P25 most likely contributes to the enhanced tonic inhibition observed in GAERS prior to AS onset, whereas the lack of any anatomo-morphological GABAergic differences in GAERS S1po suggests that functional more than structural abnormalities underlie the origin of cortical paroxysms in S1po of this AS model.

This article is part of the “Special Issue Dedicated to Norman G. Bowery”.

## Highlights

• GABA-IR profiles increase in P25 to P90 VB neuropil in GAERS but not in NEC and Wistar rats.
• NRT GABA-IR neurons increase in P25 and P90 GAERS and NEC, but not in Wistar rats.
• GABA-IR neuron density increases in S1po of GAERS at P25 and P90 and in Wistar at P90.

## 1 Introduction

Absence seizures (ASs) are a form of genetic generalized non-convulsive seizures that involve brief impairment of consciousness, occasional myoclonic jerks of the eyes and peri-oral automatisms, and spike-and-wave discharges (SWDs) in the EEG which result from paroxysmal oscillations within cortico-thalamic networks (Blumenfeld, 2005, Crunelli and Leresche, 2002). In contrast to the classical view of ASs being fully generalized from the start, recent non-invasive imaging studies in humans and intracranial electrical recordings in AS models have now shown that the paroxysmal activity initiates in localized cortical regions. Whereas in humans these regions are most often in frontal and parietal cortices, SWDs in rat genetic models of typical ASs have been shown to initiate from the peri-oral region of primary somatosensory cortex (S1po) before spreading to the ventrobasal thalamic nucleus (VB) (the somatotopic thalamic nucleus to S1) and to other cortical and thalamic areas (Meeren et al., 2002, Polack et al., 2007).

As with other forms of epilepsy, dysfunctions in the brain gamma-aminobutyric acid (GABA) system are thought to have a crucial role in the pathogenesis of ASs. In particular, many studies in human cohorts affected by these seizures have highlighted potential abnormalities related to various genes coding for proteins of the GABAergic system, including transporters, synthetizing enzymes and subunits of both GABA_A_ and GABA_B_ receptor subtypes (Crunelli and Leresche, 2002, Hirose, 2014). In-depth investigations in animals have shown that the consequences of such GABAergic abnormalities are most often selective to some regions of cortico-thalamic networks. Thus, miniature and evoked GABA_A_ synaptic currents are altered in the thalamic reticular neurons (NRT), but not in the VB or layer 2/3 cortical neurons of Generalized Absence Epilepsy Rats from Strasbourg (GAERS), a genetic model of ASs, compared to their non-epileptic control (NEC) strain (Bessaïh et al., 2006). On the other hand, mice homologous for the human AS-linked R43Q mutation of the GABA_A_R γ2 subunit (i.e., γ2 R43Q model) show reduced phasic inhibition in cortical layer 2/3 but not in NRT and VB neurons (Tan et al., 2007) and cortical layer 4 (Currie et al., 2017) compared to their wild-type littermates. Moreover, conditional ablation of CaV2.1 calcium channels in cortical somatostatin- and parvalbumin-positive GABAergic interneurons alters their GABA release, leading to increased cortical pyramidal cell excitability and generalized seizures including absences (Rossignol et al., 2013).

In sharp contrast to the loss-of-function of phasic GABA_A_R inhibition, tonic GABA_A_ current, which is mediated by extrasynaptic GABA_A_Rs, is increased in VB thalamocortical neurons of genetic rat and mouse AS models prior to seizure onset and continues to be enhanced during ASs (Cope et al., 2009, Errington et al., 2011, Errington et al., 2014). Indeed, basal extracellular GABA levels are higher in the VB (but not cortex) of GAERS compared to NEC (Richards et al., 1995). Notably, these animal data are supported by the findings that tiagabine, a GABA reuptake inhibitor, and vigabatrin, a GABA transaminase inhibitor, can exacerbate (or induce) ASs and absence status in humans (Panayiotopoulos, 1999), and by the recent observation of a selective increase in thalamic, but not cortical GABA levels in a child with ASs (Leal et al., 2016). In contrast, the tonic GABA_A_ current in VB neurons is not affected in α1 KO mice that express ASs (Zhou et al., 2015), and is drastically reduced in layer 2/3 and in VB neurons of the γ2 R43Q mouse model (Mangan et al., 2017), indicating that diverse changes in the GABAergic system can support the expression of these non-convulsive seizures.

As far as the anatomo-morphological features of GABAergic neurons in cortico-thalamic networks are concerned, a recent study has shown a higher proportion of GABAergic cells in the ventroposteromedial (VPM) and ventroposterolateral (VPL) thalamic nuclei, the two subdivision of the VB, in 6–12 month old GAERS than in age-matched normal Wistar rats (Cavdar et al., 2014), in contrast to the accepted view that in this rodent thalamic nucleus GABAergic interneurons are only sparse or absent (Arcelli et al., 1997, Harris and Hendrickson, 1987, Houser et al., 1980). Moreover, a developmental decrease in the number of NRT GABA-positive and GABA-negative neurons has been described in GAERS at postnatal day 30 (P30) to P60 compared to normal Wistar rats (Cavdar et al., 2013), whereas GABA-immunoreactive (GABA-IR) profiles in the GAERS VB shows a 3-fold increase at the same postnatal ages compared to Wistar rats (Cavdar et al., 2012). In the γ_2_R43Q mouse model, a higher density of various GABAergic interneurons has been reported in S1, though paradoxically the ratio between putative excitatory and inhibitory neurons was found to be decreased (Wimmer et al., 2015). In summary, there is no comprehensive analysis of the developmental changes of the GABAergic populations in AS-related brain regions, and as far as inbred models are concerned, these have mostly been compared with normal rat strains, a subset of which are known to express ASs (Marescaux et al., 1992).

In this study, we employed light microscopy GABA immunocytochemistry to quantify in GAERS the developmental profile of GABAergic neurons and GABA-IR profiles in the neuropil under the same experimental conditions in S1po, VB and NRT, three key regions involved in the paroxysmal oscillations that underlie ASs. We selected P15 (i.e., two days before tonic GABA_A_ inhibition starts to increase in the VB (Cope et al., 2009)), P25 (i.e., a time when full-blown ASs start to occur in GAERS), and P90 (a time when all GAERS have experienced at least 2 months of continuous ASs) (Depaulis et al., 2016). The dorsal lateral geniculate nucleus (dLGN), a sensory thalamic nucleus that differently from the VB contains local GABAergic interneurons (Parnavelas et al., 1977), and the primary motor cortex (M1), which is adjacent to S1po and whose neurons in GAERS do not show altered intrinsic electrical properties (Polack et al., 2007), were also investigated. Moreover, the GAERS data were compared to those obtained in the same brain regions of both normal Wistar rats and NEC, the Wistar-derived strain that is completely devoid of ASs (Vergnes et al., 1987).

## 2 Experimental procedures

Experiments were conducted in accordance with the UK Animals (Scientific Procedures) Act 1986, local Ethical Committee Guidelines and current recommendations for experimental work in epilepsy (Lidster et al., 2015).

### 2.1 Animals and fixation

Thirty-six rats (4 GAERS, 4 NEC and 4 Wistar, each of P15, P25 and P90) were used in this study. The animals were deeply anesthetized with a mixture (4.0 ml/kg) of sodium pentobarbital (48 mg/kg, intraperitoneally, i.p.) and chloral hydrate (40 mg/kg); i.p. and perfused intracardially using a peristaltic pump (flow rate 30–35 ml/min) as follows: 0.9% saline (+4 °C) for 2 min, followed by a solution of 4% paraformaldehyde-0.2% glutaraldehyde in 0.1 M sodium phosphate buffer, pH 7.4 (flow rate 10 ml/min) for 30 min. The brains were removed from the skull and postfixed in the final fixative for 2–4 h. The brains were then cryoprotected in 30% sucrose solution in phosphate buffered saline (PBS), pH 7.4 at + 4 °C for 48 h and cut in the coronal plane at 30 μm section thickness on a freezing sliding microtome. The sections (1 in 5 series) were stored in 30% sucrose solution in PBS at −20 °C (for immunohistochemical staining) or in 10% formalin at room temperature (for thionin staining) until processed.

### 2.2 Immunoperoxidase experiments

The free-floating coronal sections were washed three times (10 min each time) in 0.02 M PBS, pH 7.4. To eliminate endogenous peroxidase activity, the sections were treated with 1% H_2_O_2_ in H_2_O for 15–30 min, and then rinsed 6 times in 0.02 PBS. To block non-specific binding, the sections were incubated in a solution containing 10% normal goat serum (Colorado Serum Co., Denver, CO, #CS 0922) and 0.3% Triton X-100 in0.02MPBS for 2 h at room temperature. Thereafter, the sections were incubated in a solution containing mouse *anti*-GABA monoclonal antibody (diluted 1:1000, Sigma #A 0310; Saint Louis, Missouri, 63103, USA), 0.5% Triton X-100, and 1% normal goat serum for 48 h at 4 °C. Following incubation in the primary antiserum, the sections were washed three times (10 min each) in 0.02MPBS containing 2% normal goat serum. Sections were then incubated in a solution containing goat biotinylated anti-mouse (1:200, Vector, Burlingame, CA, BA-9200), 1% normal goat serum and 0.3% Triton X-100 in 0.02M PBS, pH 7.4 for 60 min at room temperature. Sections were then washed three times (10 min each) in 0.02MPBS containing 2% normal goat serum and were then transferred to avidin–biotin complex (ABC kit Vectastain, PK-6100, Vector Laboratories, Burlingame, CA) for 45 min, and the immunoperoxidase reaction was developed by 3.3′-diaminobenzidine (DAB kit, SK-4100, Vector Laboratories, Burlingame, CA). After washing, the sections were mounted onto gelatin-coated slides, dried overnight at 37 °C, defatted and intensified, according to previous evidence (Lewis et al., 1986), with OsO4 (0.005%, Electron Microscopy Sciences, #19130, Ft. Washington, PA) and thiocarbohydrazide (0.05%, Electron Microscopy Sciences, #21900), dehydrated in ethanol, cleared in xylene, and coverslipped with Entellan (Merck, Darmstaldt, Germany).

### 2.3 Specificity of antibodies

The specificity of mouse *anti*-GABA monoclonal antibody (Sigma #A 0310) has previously been characterized (Benson et al., 1992, Storm-Mathisen et al., 1983). In the present experiments, control sections incubated without the primary antibodies resulted in a complete disappearance of stained profiles. The omission as well as the replacement of the secondary antibodies with inappropriate secondary antibodies resulted in the elimination of all immunohistochemical staining.

### 2.4 Thionin staining

To help identify the boundaries of the NRT, VB, dLGN, M1 and S1po, sections adjacent to immunoperoxidase sections were stained with thionin as follows. Sections were taken out of the 10% formaldehyde solution, mounted on gelatin-coated slides and dried overnight at 37 °C. Sections were defatted 1 h in a mixture of chloroform/ethanol 100% (1:1), and then rehydrated through a graded series of ethanol, 2 × 2 min in 100% ethanol, 2 min in 96% ethanol, 2 min in 70% ethanol, 2 min in50% ethanol, 2 min in dH_2_O, and stained 30 s in a 0.125% thionin (Fisher Scientific) solution, dehydrated and coverslipped with Entellan (Merck, Darmstaldt, Germany).

### 2.5 Analysis of sections

Sections were analyzed using a Leica DMRB microscope. Brightfield images were acquired by means of a Polaroid DMC digital camera (Polaroid Corporation, Cambridge, MA, USA) and DMC 2 software. Contrast and brightness were adjusted to reflect the appearance of the labeling seen through the microscope using Adobe Photoshop CS3 Extended 10.0 software (AdobeSystems, San Jose, CA).

To calculate the density of GABAergic neurons, immunostained somata were plotted in every fifth section throughout each area with a computer-aided digitizing system (Accustage 5.1, St. Shoreview, MN). The boundaries of the NRT, VB, dLGN, M1, and S1po were drawn from the adjacent thionin-stained sections using a stereo-microscope equipped with drawing tube. The outlines were superimposed on computer generating plots using Corel Draw X3 (Corel corporation, Ottawa, Ontario, Canada). The area measurements were done from the line drawings by using AxioVision Rel.4.8 (Zeiss). The density of immunostained neurons in the different areas was calculated as number of somata/mm^2^ in each section separately. For each rat 5–10 sections were analyzed. The neuronal counts are expressed as the mean number of somata/mm^2^ ± standard error of the mean (SEM) and the data from the GAERS, NEC and Wistar rats were compared. Student's *t*-test was used to compare the statistical differences between GAERS, NEC and Wistar rats, with a significance level of P < 0.05. GABA-positive neurons and data concerning the percentage of the image covered by GABA immunostaining were determined using the automatic threshold algorithm of ImageJ (version IJ 1.46r downloaded from http://imagej.nih.gov/ij/download.html). With this algorithm, thresholding is used to extract an object from its background by assigning an intensity value “T” (threshold) for each pixel, such that each pixel is either classified as an object point or a background point, thus creating a binary image (binarization) of object points (positive immunostaining) versus background points (lack of immunostaining). An adjacent series of sections stained with thionin was used to determine the cytoarchitectonic boundaries of RTN, VB, dLGN, M1, and S1po. For this analysis images were taken using the Leica DMRB microscope under identical acquisition parameters from GAERS, NEC and Wistar rats. For each rat 5–10 sections were analyzed. All data are given as mean ± standard error of the mean (SEM) and differences between GAERS, NEC and Wistar rats were evaluated using the RMANOVA with Turkey HSD test, with a significance level at P < 0.05.

## 3 Results

### 3.1 General characteristics of GABA immunostaining

**Fig. 1:**
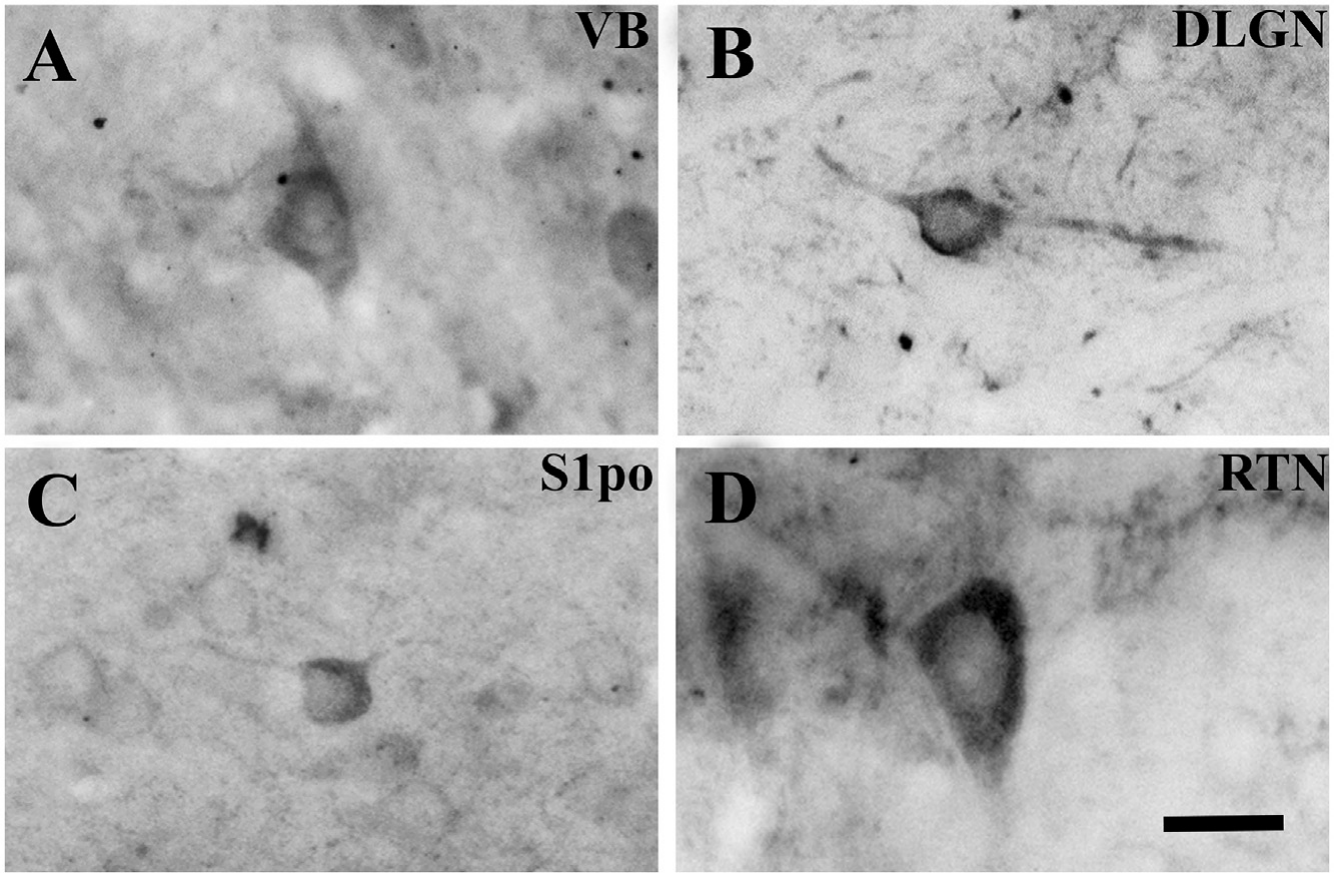
**GABA-IR neurons in different thalamic and cortical regions.** Brightfield photomicrographs of coronal sections showing representative examples of GABA-IR neurons in ventrobasal thalamus (VB) (**A**), dorsal lateral geniculate nucleus (dLGN) (**B**), peri-oral region of the primary somatosensory cortex (S1po) (**C**), and nucleus reticularis thalami (NRT) (**D**). Note that in VB, dLGN and S1po GABA-IR neurons show a multipolar (**A**,**C**) or fusiform (**B**) shape that corresponds to classical GABAergic interneurons. The GABA-IR neuron in the NRT (**D**) has typical multipolar appearance of neurons in this thalamic nucleus. Scale bar = 15 μm in **A** also applies to **B-D**.

GABA immunoreactivity was especially associated with somata and neuronal processes. GABA-IR neurons located in VB, dLGN, S1po and M1 were small, had a multipolar or fusiform shape (Fig. 1A–C) as previously described for interneurons in these different areas (Amadeo et al., 2001, Arcelli et al., 1997, Barbaresi et al., 1986, Harris and Hendrickson, 1987, Spreafico et al., 1994). In the NRT, GABA-IR neurons were fusiform, or multipolar and resembled the main cell type of this nucleus (Fig. 1D) (De Biasi et al., 1997). Neuropil immunostaining often contained axonal and dendritic processes, as well as GABA-positive puncta resembling axon terminals.

### 3.2 Ventrobasal thalamus (VB)

**Fig. 2:**
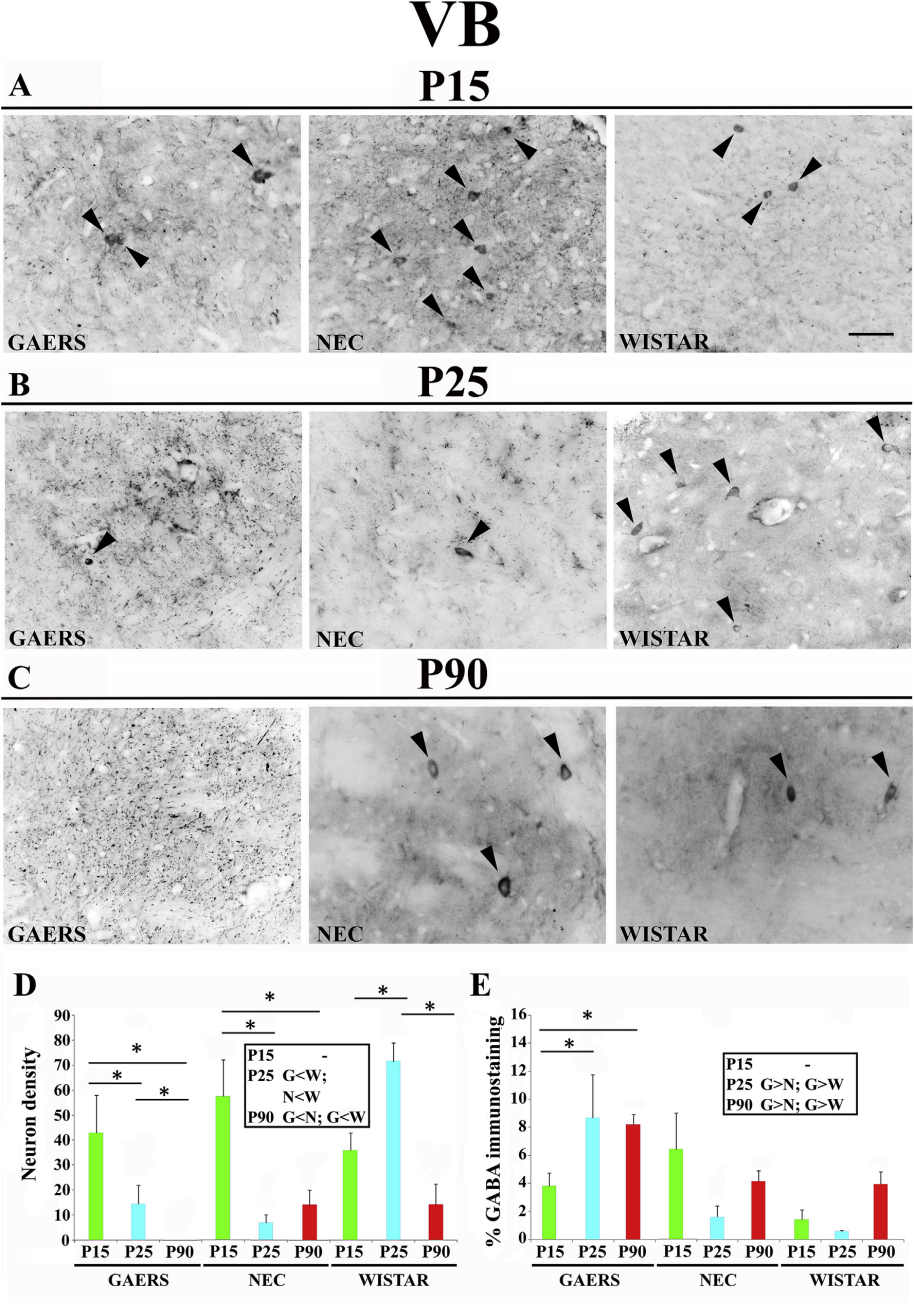
**Developmental profile of GABA immunoreactivity in the ventrobasal thalamus of GAERS, NEC and WISTAR rats.** **A**, **B**, **C** show typical brightfield photomicrographs of GABA immunoreactivity in coronal sections of the ventrobasal (VB) thalamus of GAERS, NEC and WISTAR rats at P15, P25 and P90. Arrowheads indicate GABA-IR neurons. (**D)** Histograms showing the density (neurons/mm^2^ ± SEM) of GABA-IR neurons in the VB at the indicated postnatal days in the 3 strains. (**E)** Histograms of percentage area (±SEM) covered by GABA immunoreactivity at the indicated postnatal days in the 3 strains. In **D** and **E**, * indicates P < 0.05 (ANOVA for repeated measures followed by a post-hoc Turkey HSD test). Insets in **D** and in **E** show statistically significant differences between GAERS (G), NEC (N) and Wistar (W) at the indicated postnatal days, with > and < indicating a higher and smaller level, respectively. Scale bar = 40 μm in **A** also applies to **B** and **C**.

In the GAERS VB, the density (neurons/mm^2^) of GABA-IR neurons progressively and markedly decreased from P15 (43 ± 21.2) to P25 (14.5 ± 10.51) and P90 (0 ± 0; p < 0.05 for both) (Fig. 2A–D). In the NEC VB, there was a marked decrease from P15 (57.5 ± 20.5) to P25 (7 ± 4.43; p < 0.05) but no further decrease at P90 (14.2 ± 11.8; p > 0.05) (Fig. 2A–D). Differently from the findings in these two strains, in the Wistar VB the density of GABA-IR neurons first increased from P15 (36 ± 9.9) to P25 (70.5 ± 10.5; p < 0.05) and then decreased at P90 (14.3 ± 14; p < 0.05) to the same level as at P15 (Fig. 2A–D). Comparison among the 3 strains showed no difference at P15, whereas at P25 the density of GABA-IR neurons was similar in GAERS and NEC but smaller than in Wistar (Fig. 2D, inset). At P90, there were no GABA-IR neurons in GAERS and a similar small number in NEC and Wistar rats (Fig. 2D, inset**)**.

No developmental change in the area covered by GABA-IR neuropil was detected within each of the GAERS, NEC and Wistar strain (Fig. 2A–C) except in GAERS between P15 and P90. Moreover, no difference was present among the 3 strains at P15 (Fig. 2A–D, inset). Notably, however, GAERS GABA-IR neuropil was larger than in NEC and Wistar at both P25 and P90 (Fig. 2A–E, inset).

### 3.3 Nucleus reticularis thalami (NRT)

**Fig. 3:**
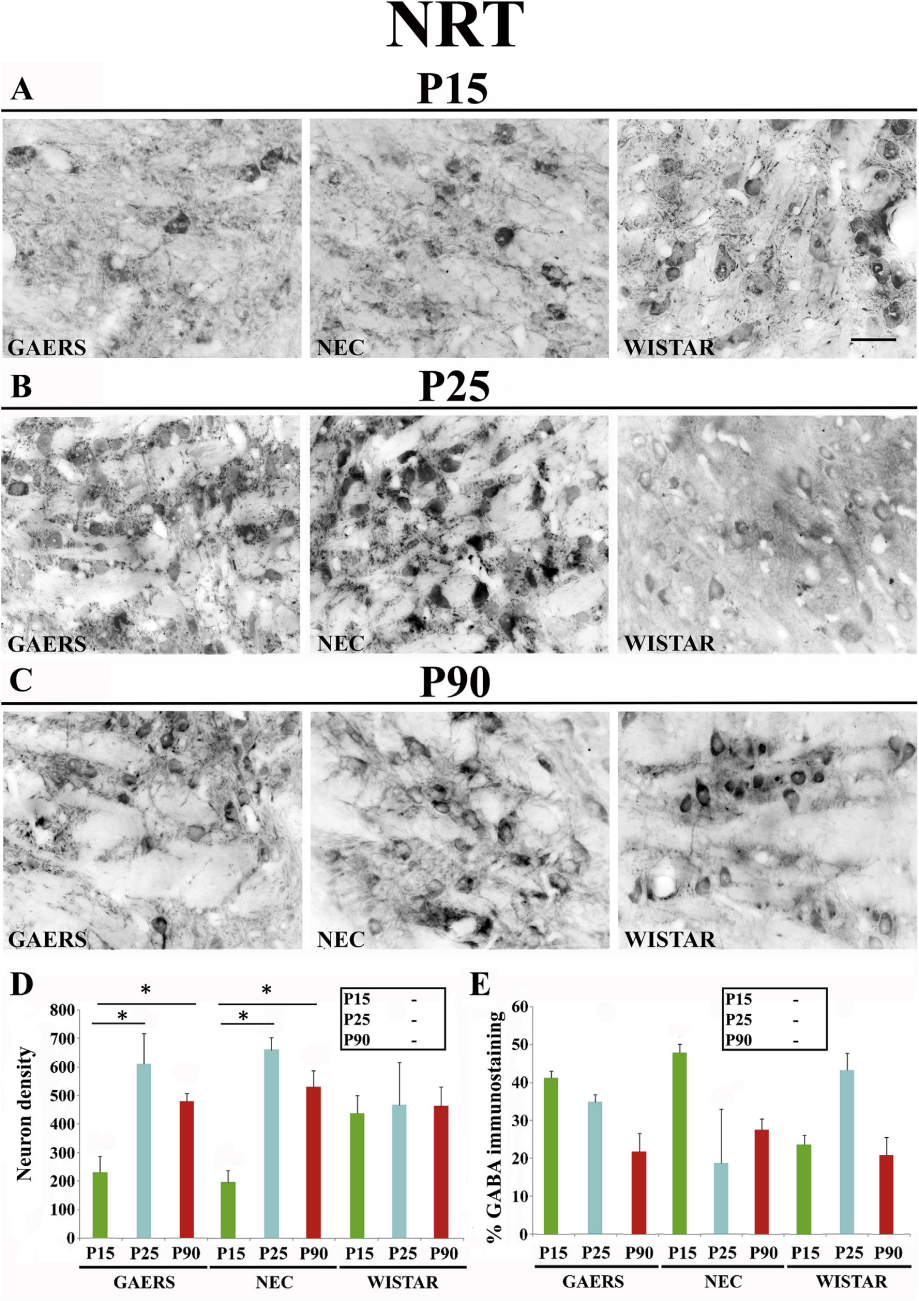
**Developmental profile of GABA immunoreactivity in the nucleus reticularis thalami of GAERS, NEC and WISTAR rats.** **A**, **B**, **C** show typical brightfield photomicrographs of GABA immunoreactivity in coronal sections of the nucleus reticularis thalami (NRT) of GAERS, NEC and WISTAR rats at P15, P25 and P90. (**D)** Histograms showing the density (neurons/mm^2^ ± SEM) of GABA-IR neurons in the VB at the indicated postnatal days in the 3 strains. (**E)** Histograms of percentage area (±SEM) covered by GABA immunoreactivity at the indicated postnatal days in the 3 strains. In **D** and **E**, * indicates P < 0.05 (ANOVA for repeated measures followed by a post-hoc Turkey HSD test). Insets in **D** and in **E** show statistically significant differences between GAERS (G), NEC (N) and Wistar (W) at the indicated postnatal days, with > and < indicating a higher and smaller level, respectively. Scale bar = 40 μm in **A** also applies to **B** and **C**.

The density of NRT GABA-IR neurons in GAERS and NEC markedly and similarly increased from P15 (229 ± 81.3 and 198 ± 40.7, respectively) to P25 (609 ± 152 and 660 ± 62.6; respectively, p < 0.05 for both) but remained at this level at P90 (478 ± 72.3 and 530 ± 114, respectively, p > 0.05 for both) (Fig. 3A–D). No developmental changes in GABA-IR neurons was found in Wistar (Fig. 3A–D), and no difference was detected at each postnatal age among the 3 strains (Fig. 3D, inset). Moreover, no intra-and inter-strain differences were detected in the level of GABA-IR neuropil (Fig. 3A–E, inset).

### 3.4 Dorsal lateral geniculate nucleus (dLGN)

**Fig. 4:**
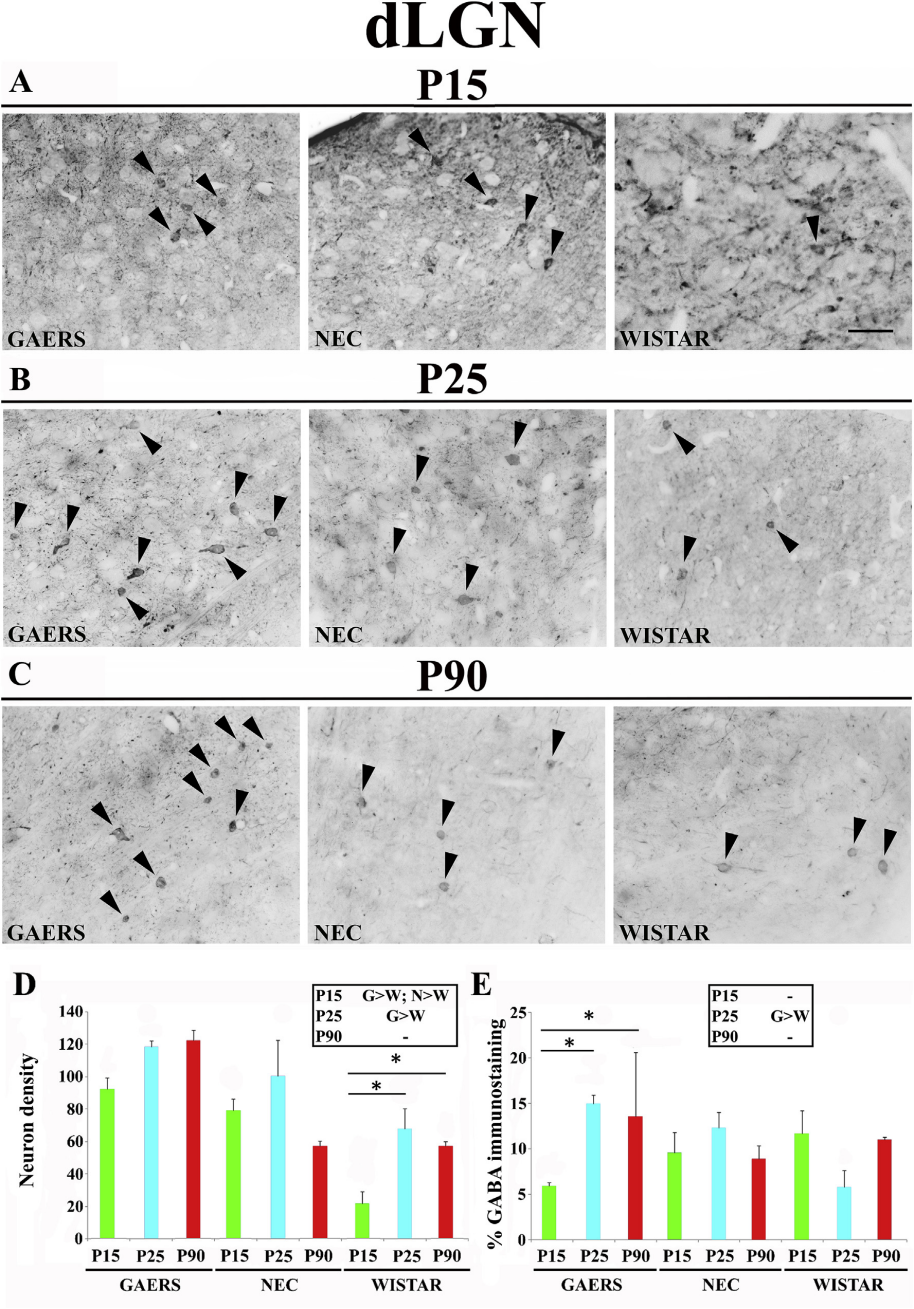
**Developmental profile of GABA immunoreactivity in the dorsal lateral geniculate nucleus of GAERS, NEC and WISTAR rats.** **A**, **B**, **C** show typical brightfield photomicrographs of GABA immunoreactivity in coronal sections of the odrsal lateral geniculate nucleus (dLGN) of GAERS, NEC and WISTAR rats at P15, P25 and P90. Arrowheads indicate GABA-IR neurons. (**D)** Histograms showing the density (neurons/mm^2^ ± SEM) of GABA-IR neurons in the VB at the indicated postnatal days in the 3 strains. (**E)** Histograms of percentage area (±SEM) covered by GABA immunoreactivity at the indicated postnatal days in the 3 strains. In **D** and **E**, * indicates P < 0.05 (ANOVA for repeated measures followed by a post-hoc Turkey HSD test). Insets in **D** and in **E** show statistically significant differences between GAERS (G), NEC (N) and Wistar (W) at the indicated postnatal days, with > and < indicating a higher and smaller level, respectively. Scale bar = 40 μm in **A** also applies to **B** and **C**.

No developmental change occurred in the dLGN GABA-IR neuron density of both GAERS and NEC (Fig. 4A–D). In contrast, in Wistar the number of GABA-IR neurons increased from P15 (21.5 ± 10.6) to P25 (68 ± 24.4, p < 0.05) and remained at this level at P90 (57 ± 3.9; p > 0.05) (Fig. 4A–D). Inter-strain comparison showed that at P15 and P25 more GABA-IR neurons were present in GAERS (93 ± 9.9 and 118 ± 7, respectively) than in Wistar (21.5 ± 10.6 and 68 ± 24.4, respectively; p < 0.05 for both) (Fig. 4D, inset). NEC GABAergic neurons density (79 ± 9.9) was significantly higher than in Wistar (21.5 ± 10.6; p < 0.05) only at P15 (Fig. 4D, inset).

The area of GABA-IR neuropil show no developmental change in NEC and Wistar, while in GAERS it increased from P15 to P25 and then remained stable at P90 (Fig. 4A–D). No inter-strain differences were observed in GABA-IR neuropil except for a larger area in GAERS than in Wistar at P25 (p < 0.05) (Fig. 4E, inset).

### 3.5 Peri-oral region of primary somatosensory cortex (S1po)

**Fig. 5:**
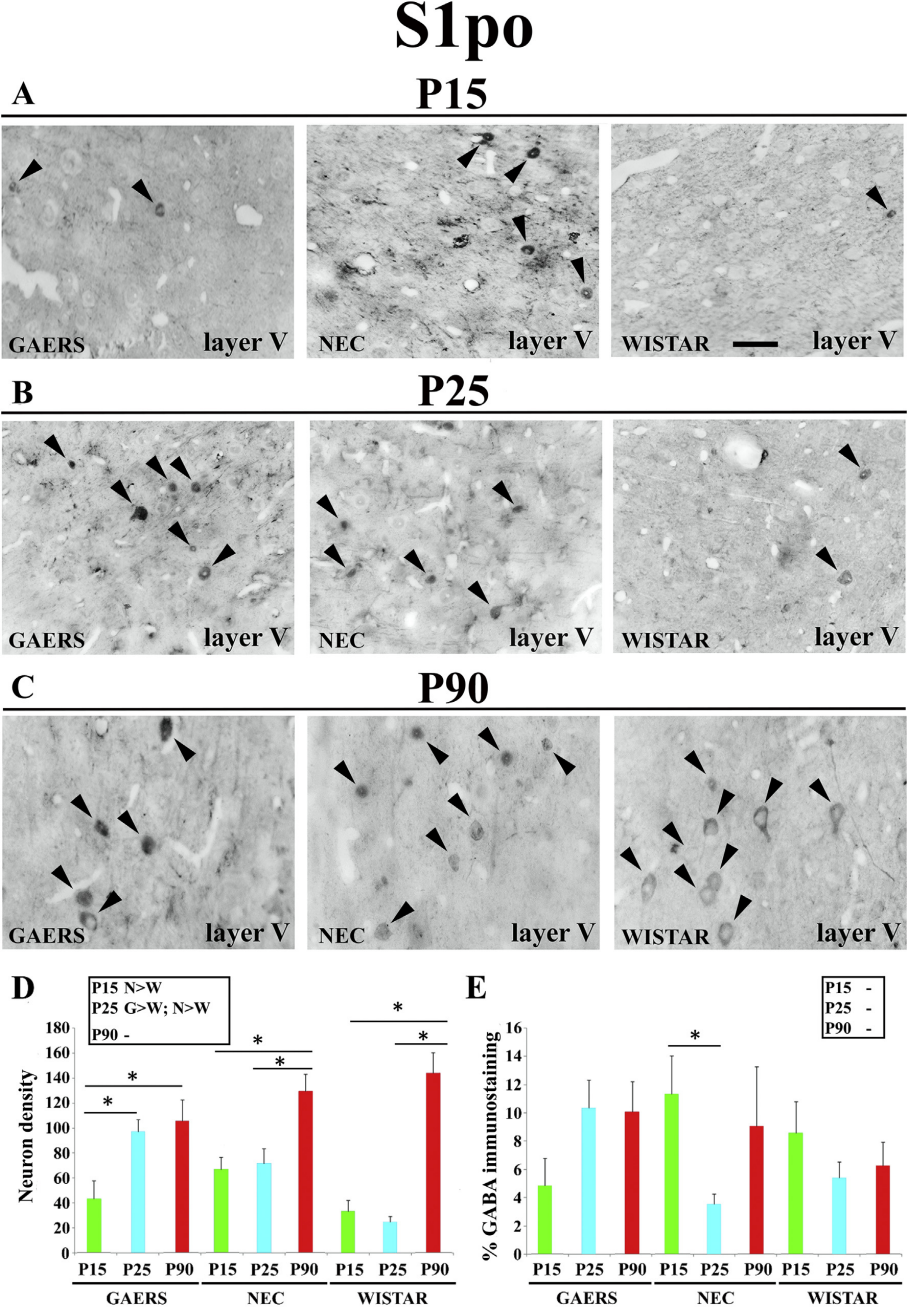
**Developmental profile of GABA immunoreactivity in the peri-oral region of primary somatosensory cortex of GAERS, NEC and WISTAR rats.** **A**, **B**, **C** show typical brightfield photomicrographs of GABA immunoreactivity in coronal sections of layer V of the peri-oral region of the primary somatosensory cortex (S1po) of GAERS, NEC and WISTAR rats at P15, P25 and P90. Arrowheads indicate GABA-IR neurons. (**D)** Histograms showing the density (neurons/mm^2^ ± SEM) of GABA-IR neurons in the VB at the indicated postnatal days in the 3 strains. (**E)** Histograms of percentage area (±SEM) covered by GABA immunoreactivity at the indicated postnatal days in the 3 strains. In **D** and **E**, * indicates P < 0.05 (ANOVA for repeated measures followed by a post-hoc Turkey HSD test). Insets in **D** and in **E** show statistically significant differences between GAERS (G), NEC (N) and Wistar (W) at the indicated postnatal days, with > and < indicating a higher and smaller level, respectively. Scale bar = 40 μm in **A** also applies to **B** and **C**.

Since no intra- or inter-strain statistically significant difference was observed between layer 5 and 6 of S1po in both the density of GABA-IR neurons and the area of GABA-IR neuropil, the data from these two layers were combined in the following analysis. The number of GABA-IR neurons in GAERS markedly increased from P15 (43 ± 25.6) to P25 (97.4 ± 21.1; p < 0.05) but remained at this level at P90 (105 ± 38.2; p > 0.05) (Fig. 5A–D). In NEC, the density was similar between P15 (66.8 ± 23.3) and P25 (71.8 ± 26.5, p > 0.05) but increased at P90 (129 ± 19.8, p < 0.05) (Fig. 5A–D). In Wistar, GABA-IR neuron density did not change between P15 (33.5 ± 21.8) and P25 (24.9 ± 12.4; p > 0.05) but increased sharply at P90 (143 ± 36.6; p < 0.05) (Fig. 5A–D, inset). Inter-strain analysis showed that at P15 GABA-IR neuron density was different only between NEC (66.8 ± 23.3) and Wistar (33.5 ± 21.8; p < 0.05) (Fig. 5D, inset). At P25, both GAERS (97.4 ± 21.1) and NEC (71.8 ± 26.6) had more GABA-IR neurons than Wistar rats (24.8 ± 12.4; p < 0.05 for both) whereas at P90 no differences among the 3 strain was observed (Fig. 5D, inset).

No developmental change was observed in the area of GABA-IR neuropil (except for a drop in NEC at P25) and there was no inter-strain difference (Fig. 5A–E, inset). Similar pattern of results was obtained among the different strains in S1po layers 2, 3 and 4 for both neuronal density and GABA-IR neuropil (see Table 1).

**Table 1:** Density of GABA-IR neurons and GABA immunostaining area in the S1po and M1 of GAERS, NEC and WISTAR rats.

S1po			
NEURONAL DENSITY			
Age	GAERS	NEC	WISTAR
LAYERS 2, 3,4			
P15	44.2 ± 9.3	69.3 ± 21.7§	34.3 ± 10.1§
P25	99.2 ± 23.2°	73.2 ± 21.7§	26.2 ± 9.3°§
P90	109 ± 31.8	133 ± 25.1	146 ± 37.2
% GABA IMMUNOSTAINING			
LAYERS 2, 3, 4			
P15	5.7 ± 1.2	11.9 ± 3.2	9.2 ± 2.7
P25	11.4 ± 2.7	5.6 ± 1.9	6.1 ± 2.1
P90	11.3 ± 3.2	10.1 ± 2.7	7.3 ± 2.1
M1			
NEURONAL DENSITY			
	GAERS	NEC	WISTAR
LAYERS 2, 3, 4			
P15	31.3 ± 7.3*	76.1 ± 19.6*§	30.7 ± 10.7§
P25	117 ± 18.1°	137 ± 33.9§	51.3 ± 9.3°§
P90	120 ± 21.3*	41.7 ± 10.1*§	110 ± 27.8§
% GABA IMMUNOSTAINING			
LAYERS 2, 3, 4			
P15	10.7 ± 2	10.1 ± 2.3	5.9 ± 1.9
P25	8 ± 2.2	4.3 ± 1	1.7 ± 1.9
P90	4.3 ± 1.1	9.1 ± 2.1	6.9 ± 1.9

Density (number of neurons/mm^2^ ± SEM) of GABA-immunoreactive somata and average percentage (±SEM) of the image area covered by GABA immunostaining in the peri-oral region of the primary somatosensory cortex (S1po) and the primary motor cortex (M1) of GAERS, NEC and WISTAR rats at P15, P25 and P90 in layers 2 to 4. *p < 0.05, GAERS versus NEC; §p < 0.05 , NEC versus WISTAR; °p < 0.05, GAERS versus WISTAR (*t*-tetst).

### 3.6 Primary motor cortex (M1)

**Fig. 6:**
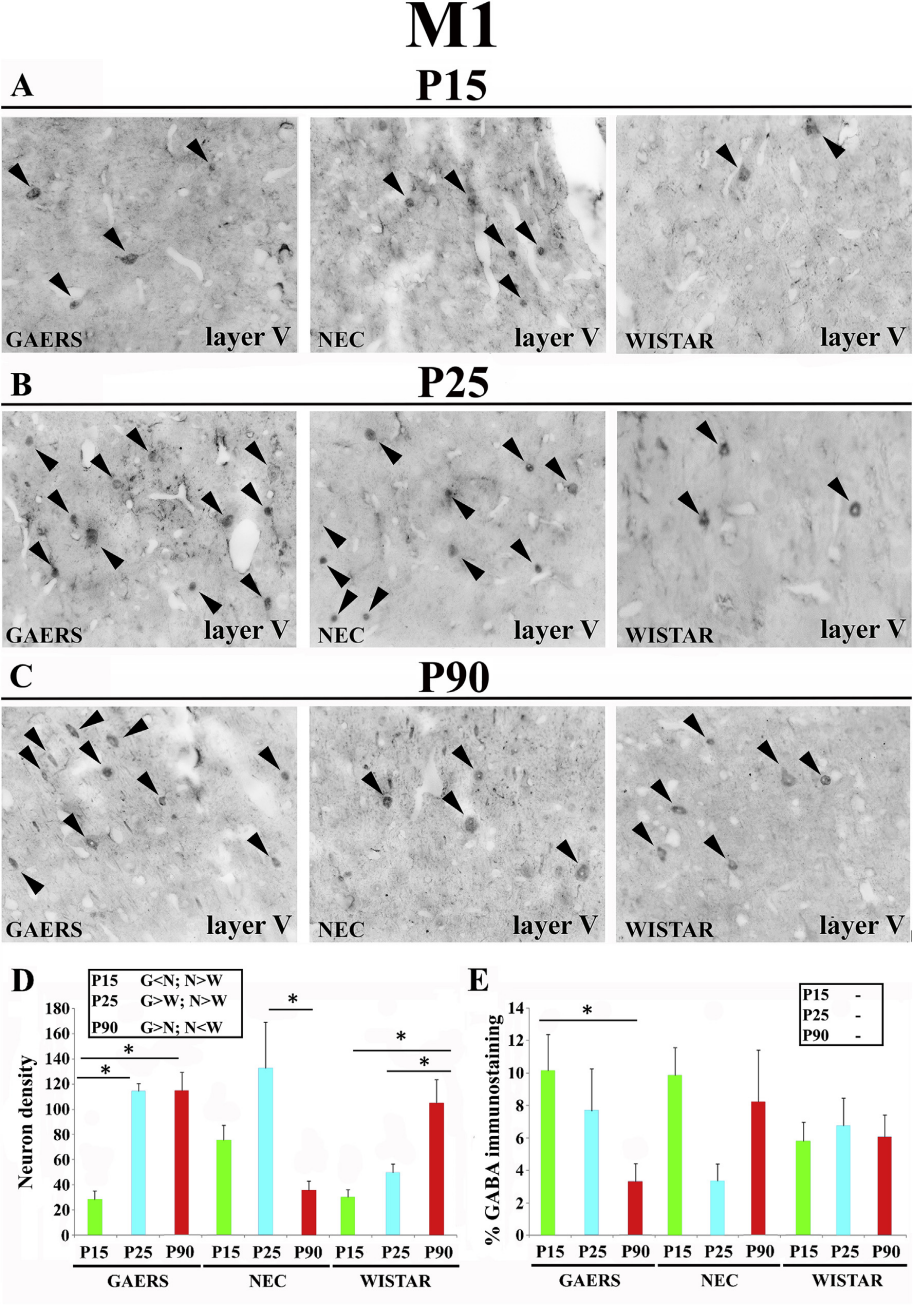
**Developmental profile of GABA immunoreactivity in the primary motor cortex of GAERS, NEC and WISTAR rats.** **A**, **B**, **C** show typical brightfield photomicrographs of GABA immunoreactivity in coronal sections of layer V of the primary motor cortex (M1) of GAERS, NEC and WISTAR rats at P15, P25 and P90. Arrowheads indicate GABA-IR neurons. (**D)** Histograms showing the density (neurons/mm^2^ ± SEM) of GABA-IR neurons in the VB at the indicated postnatal days in the 3 strains. (**E)** Histograms of percentage area (±SEM) covered by GABA immunoreactivity at the indicated postnatal days in the 3 strains. In **D** and **E**, * indicates P < 0.05 (ANOVA for repeated measures followed by a post-hoc Turkey HSD test). Insets in **D** and in **E** show statistically significant differences between GAERS (G), NEC (N) and Wistar (W) at the indicated postnatal days, with > and < indicating a higher and smaller level, respectively. Scale bar = 40 μm in **A** also applies to **B** and **C**.

Since no intra- or inter-strain statistically significant difference was observed between layer 5 and 6 of M1 in both the density of GABA-IR neurons and the area of GABA-IR neuropil, the data from these two layers were combined in the following analysis. The number of GABA-IR neurons in GAERS markedly increased from P15 (28.7 ± 13.3) to P25 (114 ± 11.8; p < 0.05) but remained at this level at P90 (114 ± 20.5; p > 0.05) (Fig. 6A–D). In contrast, in NEC no developmental change occurred between P15 (75.2 ± 24.3) and P25 (132 ± 72.3; p > 0.05) but a decrease was observed at P90 (36 ± 9.9; p < 0.05) (Fig. 6A–D). In Wistar, GABA-IR neuron density increased from P15 (30.5 ± 15.9) to P25 (50 ± 9.9; p < 0.05) but remained at this level at P90 (105 ± 56.5; p > 0.05) (Fig. 6A–D). Inter-strain analysis showed significantly higher GABA-IR neurons in NEC than in GAERS and Wistar at P15 (Fig. 6A–D, inset), whereas at P25 more GABA-IR neurons were present in GAERS and NEC than in Wistar (Fig. 6A–D, inset). At P90, the density of GABA-IR neurons was significantly higher in GAERS and Wistar than in NEC rats (Fig. 6A–D, inset).

The area of GABA-IR neuropil significantly decreased from P15 to P90 in GAERS, whereas no developmental change occurred in NEC and Wistar, and no inter-strain difference was observed at any of the postnatal days examined (Fig. 6-A-E, insert). Similar pattern of results was obtained among the different strains in M1 layers 2, 3 and 4 for both neuronal density and GABA-IR neuropil (see Table 1).

## 4 Discussion

The main findings of this study are: i) the increase in GABA-IR profiles in the VB neuropil at P25 to P90 in GAERS but not in NEC and Wistar rats; ii) the increase in NRT GABA-IR neurons in GAERS and NEC, but not Wistar rats, at both P25 and P90; and iii) the intra-species increase in GABA-IR neuron density in S1po of GAERS at P25 and P90 and in Wistar at P90.

### 4.1 Methodological considerations

Three methodological issues should be considered before discussing the differences between our findings and those of previous studies in GAERS. First, we used a GABA monoclonal antibody whereas previous investigations in GAERS have employed polyclonal antibodies (Cavdar et al., 2013, Cavdar et al., 2014, Cavdar et al., 2012). Second, different GAERS colonies, though all derived from the original strain in Strasbourg (Depaulis et al., 2016, Vergnes et al., 1987), show diverse AS phenotypes, potentially resulting from different lengths of inbreeding and environmental conditions at diverse establishments (Powell et al., 2014). Third, morphological and behavioural differences have been described between NEC and Wistar rats. The NEC strain has been inbred from the same initial Wistar rat colony as the GAERS by originally selecting rats that showed no ASs (Depaulis et al., 2016, Vergnes et al., 1987). It is possible that alongside the “exclusion” of genes predisposing to the AS phenotype, other genes have been selected in NEC rats that are not directly responsible for the lack of ASs in this strain (Powell et al., 2014). Indeed, NEC rats weigh less (Fauvelle et al., 2015, Marques-Carneiro et al., 2014, Powell et al., 2014), have a smaller whole brain volume, a larger amygdala, a reduced night-time locomotor activity, a stronger anxiety-like behaviour in the open field and elevated plus-maze (Marques-Carneiro et al., 2014), and a different metabolic profile (Fauvelle et al., 2015) than Wistar rats. Therefore, these data suggest that NEC rats may not be an appropriate or sufficient control strain for GAERS and indicates that normal Wistar rats should also be used in comparative studies. In this respect, our present results further the available evidence by showing that, compared to Wistar rats, NEC have fewer GABA-IR neurons at P25 in the VB and at P90 in M1 but more GABA-IR neurons in dLGN at P15, and in S1oP and M1 at both P15 and P25.

### 4.2 GABA-IR in thalamus

The lack of GABA-IR neurons (i.e., local interneurons) in the VB of adult GAERS observed in the present study is consistent with previous evidence in adult rodents showing that thalamic nuclei other than the dLGN are almost devoid of GABAergic interneurons (Amadeo et al., 2001, Arcelli et al., 1997, Barbaresi et al., 1986, Harris and Hendrickson, 1987, Spreafico et al., 1994). Our findings, however, are in contrast with the results of a recent study that reported a higher density of GAERS GABA-IR neurons in VPM (14.9%) and VPL (11.1%) compared to Wistar rats (Cavdar et al., 2014). Since, in agreement with these authors we found a similar density of GABA-IR neurons in Wistar rats and NEC rats (about 13/mm^2^), the difference in GABA-IR neurons in GAERS VB may be related to different colonies used in these studies and most likely have no major role in the expression of ASs.

The marked developmental increase in GABA-IR neuropil in the GAERS, but not in Wistar and NEC, VB agrees with the data from an electron microscope study showing a high number of GABAergic F1 axon terminals in the adult GAERS VB (Cavdar et al., 2012). Most likely, this increase of GABA-IR neuropil may result from the higher number of GABA-IR neurons in GAERS NRT, leading in turn to an increased density of axonal branching/arborization. However, it cannot be excluded that an increased GABAergic input from other thalamic or extra-thalamic sources, i.e. zona incerta (Bartho et al., 2002, Bodor et al., 2008, Bokor et al., 2005) substantia nigra, anterior pretectal nucleus (Bodor et al., 2008, Ilinsky et al., 1997) is responsible for the high level of GABA-IR neuropil in the GAERS VB.

In contrast to the VB, no developmental reduction of GABA-IR neurons was observed in the dLGN of both GAERS and NEC, while a clear increase was observed in Wistar dLGN from P15 up to P90 (as in Wistar VB). Developmental changes in GABA-IR neuropil were similar in VB and dLGN, with GAERS showing an increase while no changes were present in NEC and Wistar. In the absence of any functional data on interneuron-dependent inhibition in the GAERS LGN, it is difficult to relate any of these differences to the mechanism by which this sensory thalamic nucleus contributes to the expression of ASs in GAERS.

A developmental increase in the number of the GABA-IR neurons in GAERS and NEC, but not in the Wistar, NRT was observed in this study from P15 to P25-P90. Other authors have shown a similar increase at P10 to P20 in both Wistar and GAERS but a progressive decrease at P60 only in GAERS (Cavdar et al., 2013), while a reduction in Wistar at both P90 and P720 in Wistar (Ramos et al., 1995). As it does occur in both GAERS and NEC, it is unlikely that the increased density of NRT GABA-IR neurons in GAERS during development is related to the expression of the absence phenotype.

In summary, the increased axonal branching/arborization that underlie the higher levels of GABA-IR neuropil observed in the VB of GAERS but not Wistar and NEC, is the most likely anatomo-morphological difference in the thalamic GABAergic-system among the 3 rat strains examined that can be linked to the increase in GABA release (Cope et al., 2009, Richards et al., 1995, Sutch et al., 1999). This increase in arborization before absence seizure onset reported here would exacerbate the GAT-1 impairment that is known to be present in GAERS (Cope et al., 2009) and may have epileptogenic significance. The increase in neuronal hyperpolarisation and membrane conductance that occurs following enhanced tonic GABA-A conductance in thalamocortical neurons (Cope et al., 2005), due to a combination of NRT hyper-innervation (present findings) and reduced GABA uptake by GAT-1 (Cope et al., 2009), would most likely alters the finely tuned excitation-inhibition balance within thalamo-cortical networks (Bright et al., 2007). In particular, the responsiveness of TC neurons to excitatory sensory synaptic input will be reduced, ‘gating’ information flow through the thalamus, and leading in the case of absence seizures to a reduced ability to respond to external stimuli (Blumenfeld, 2005).

### 4.3 GABA-IR in cortex

We observed from layers 2 to 6 a developmental increase of GABA-IR neurons in GAERS S1po at both P25 and P90 whereas in NEC and Wistar it only occurred at P90. However, at none of the times examined was the density in GAERS different from both NEC and Wistar. Moreover, no major differences were present in the level of GABA-IR neuropil among the 3 strains in both S1po and M1. Therefore, it is unlikely that any of the anatomo-morphological findings observed in GAERS cortex in the present study contributes to the pathophysiological mechanisms that lead to SWD appearing first in S1po of this absence model. Indeed, no difference in the properties of cortical GABA_A_ IPSC in layer 2/3 of GAERS and NEC has been found (Bessaïh et al., 2006), and inhibitory mechanisms will need to be investigated in cortical layer 5/6 where the main functional abnormality of GAERS seems to reside (Polack et al., 2007).

Similarly to our data, a higher density of cortical GABA-IR neurons (both parvalbumin-, calbindin- and calretin-positive cells) has been reported in S1 of the γ2R43Q mouse model of ASs, particularly in layers 2/3 and 5/6, at P35 though older animals were not investigated (Wimmer et al., 2015). Differently from GAERS, however, cortical GABA_A_ IPSC properties are altered (Tan et al., 2007) and the functional connectivity of fast-spiking interneurons is reduced (Currie et al., 2017) in these knock-in mice. Alterations of cortical interneuron function has also been reported in stargazer mice, another model of ASs, where a marked reduction in GluA4 expression level in cortical parvalbumin-positive interneurons leads to a paradoxical pro-epileptic effect of NMDA antagonists (Maheshwari et al., 2013). Notably, transplantation of medial ganglionic eminence cells into primary visual cortex of stargazer mice rescues their absence phenotype (Hammad et al., 2014).

## 5 Conclusions

Our study indicates that the number of GABAergic neuron in the VB of GAERS does not increase during development, and therefore the enhanced GABA-IR neuropil in this nucleus most likely results from an increased arborization of NRT GABAergic fibers rather than an increased density of GABA-IR neurons in the NRT. This increased GABAergic innervation is present at P25, i.e. before ASs are fully developed, and may contribute to the enhanced tonic inhibition observed in GAERS in this postnatal time period. Unexpectedly, no major anatomo-morphological differences were observed in GAERS M1 and S1po compared to both NEC and Wistar rats, suggesting that functional more than structural abnormalities underlie the cortical paroxysms in GAERS. Finally, the presence of key differences in GABA-IR neuron and neuropil in cortex and thalamus between NEC and Wistar rats further supports the need to include both these strains as control animals of GAERS.

## Conflicts of interest

The authors declare no competing financial interests.
